# Objective drivers of subjective well-being in geriatric inpatients: mobility function and level of education are general predictors of self-evaluated health, feeling of loneliness, and severity of depression symptoms

**DOI:** 10.1007/s11136-016-1355-x

**Published:** 2016-07-07

**Authors:** Barbara Bień, Katarzyna Bień-Barkowska

**Affiliations:** 1 Department of Geriatrics, Medical University of Bialystok, Fabryczna 27, 15-471 Bialystok, Poland; 2 Institute of Econometrics, Warsaw School of Economics, Madalińskiego 6/8, 02-510 Warsaw, Poland

**Keywords:** Self-evaluated health, Feeling of loneliness, Depression, Geriatric inpatients, Timed Up and Go test, Comprehensive geriatric assessment

## Abstract

**Purpose:**

Identification of optimal predictors for different indicators of subjective well-being (SWB) in geriatric inpatients: (1) self-evaluated health status (SEH), (2) feeling of loneliness (FoL), and (3) severity of depression symptoms (SoDS). Investigation of the relationship between response categories of the SWB indicators and their predictors.

**Methods:**

The data were collected retrospectively from hospital records. All 555 geriatric inpatients underwent a comprehensive geriatric assessment, including the Timed Up and Go (TUG) test. The Bayesian information criterion was applied in ordinal logistic regression models to identify optimal predictors of SEH, FoL, and SoDS among different objective factors.

**Results:**

After controlling for high-stress situations in the recent past, motor slowness measured with the TUG test, and a level of education were jointly selected as the best predictors of all three SWB indicators. The speed of performing the TUG test improved SEH (OR = 2.08) and decreased both FoL (OR = 0.41) and SoDS (OR = 0.41). A higher level of education improved SEH (OR = 1.05) and alleviated both FoL (OR = 0.96) and SoDS (OR = 0.92). Additionally, a higher level of SEH was positively correlated with a lower BMI, improved instrumental activities of daily living (I-ADL), and higher hemoglobin level. FoL was reinforced by the level of comorbidity, and SoDS was increased by impaired basic ADL.

**Conclusion:**

Although SWB in geriatric inpatients can be explained by objective comorbidities and disabilities, the good motor function (i.e., a TUG test outcome of less than about 20 s) and a higher level of education were the general predictors that exert an independent beneficial impact on all three SWB indicators.

## Introduction

Quality of life (QoL) is an umbrella notion that encompasses a plethora of objective and nonobjective factors “that influence what we value in living, reaching beyond its material side” [1]. Whereas objective elements of QoL (e.g., health, employment, income, skills or education, social connections) are measurable—or at least confirmable by others—the subjective dimension of QoL [i.e., subjective well-being (SWB)] measures how people evaluate the quality of their lives on an internal scale, both cognitively and emotionally [2].

According to the Organisation for Economic Co-operation and Development (OECD), SWB is defined as “good mental states, including all of the various evaluations, positive and negative, that people make of their lives and the affective reactions of people to their experiences” [3]. The complex and multidimensional concept of SWB covers three elements: life evaluation (a person’s reflective thoughts about life, life satisfaction); affect (a person’s feelings or moods—happiness, sadness, anger, stress); and eudaimonia (a sense of meaning and purpose in life) [3, 4]. Although all of these aspects refer to the general self-evaluation of life, only affect—also known as hedonic well-being—expresses internally experienced feelings or intrinsic emotional states that refer to a particular moment in time (e.g., current self-evaluation of health, feeling of loneliness, depressive mood). This element of SWB is largely associated with the subject matter of this article.

Positive emotions can trigger the restoration of an individual’s well-being, an improvement in their broad-minded coping skills, and can extend the individual’s scope of attention and cognition [5]. These emotions promote longevity [6–8] and reduce the risk of stroke and other medical conditions [9, 10], and depression symptoms predict negative health outcomes in older adults [11]. Essentially, the will-to-live and optimism predict higher survival rates among older people [12] and lower feelings of loneliness [13].

Geriatric inpatients are usually the most comorbid and the most complicated of all medical cases. They are generally portrayed as the “oldest, sickest, most complicated and frail” inpatients [14]. Accordingly, multifaceted measurement and operationalization of their SWB is a very challenging task in everyday clinical routine. Clinicians usually question their patients about various afflictions and complaints but do not investigate current emotional states of their patients in a structured and in-depth manner, including their self-evaluation of health (SEH), feelings of loneliness (FoL), or the severity of their depression symptoms (SoDS). Although a comprehensive geriatric care includes an assessment of emotions along with functional status and health conditions in older adults, even geriatricians very rarely reach beyond the standard diagnostics of depression. However, it is known that the positive self-perception of aging may mitigate the negative impact of comorbidity on the quality of life [15].

Studies of SWB and objective measures of health in older people usually collect data from general population surveys [16–18], nursing homes [19], or patients with single diseases [20, 21], explaining the scarcity of knowledge about objective predictors of SWB in a heterogeneous and comorbid population of geriatric inpatients. Moreover, little is known about objective drivers (i.e., objective sociodemographic and health-related factors) that explain internally experienced feelings or emotional states within the same group of patients with multiple impairments and chronic diseases. Accordingly, the main objective of this study is to identify the optimal combination of independent objective predictors for each single indicator of SWB (SEH, FoL, and SoDS) in geriatric inpatients. This article also investigates the relationships between the selected categories of dependent variables (SEH, FoL, and SoDS) and their objective drivers.

## Methods

### Study design

The data were collected during 2013 from 555 consecutive patients (mean age 81.0, SD ± 7.2) admitted to the geriatric ward of a medium-sized hospital that draws from a population of over 300,000. No exclusion criteria were applied. Retrospective data for the study were retrieved from the patients’ medical records, including patient interviews, laboratory reports, and elements of the comprehensive geriatric assessment (CGA) that provide the most complete interdisciplinary diagnostic instruments for the identification of health problems in older people [22].

A geriatric inpatient was defined as a person of advanced old age, with complex morbidity, and in need of a CGA due to some recent deterioration of health and a high risk of death in the next 2 years according to the VES-13 scale [23]. The CGA was routinely carried out in person by the geriatric team (i.e., geriatricians, nurses, and physiotherapists) on the first or second day after each patient’s admission. If an inpatient was unable to answer a question, the corresponding item of the questionnaire was considered missing and was excluded from further analysis. If an inpatient was unable to perform a task from the CGA, the tasks scores were adjusted to account for the unperformed tasks.

### Measures of subjective well-being as dependent variables

SEH was assessed using the question “How do you evaluate your health status in comparison with other people at your age?” (answer options: significantly worse, moderately worse, moderately better, significantly better). FoL was evaluated with the question “Do you feel lonely?” (answer options: never, sometimes, often). The SoDS was assessed using the 15-item Geriatric Depression Scale [24] (GDS) with a range of 0–4 indicating no depression, 5–8 indicating mild depression, 9–11 indicating moderate depression, and 12–15 indicating severe depression (Table 1).

**Table 1:** Characteristics of geriatric inpatients (n = 555)

	Missing cases	*n*	%
Self-evaluated health	29		
Significantly better		15	2.9
Moderately better		190	36.1
Moderately worse		233	44.3
Significantly worse		88	16.7
Feeling loneliness	22		
Never		189	35.5
Sometimes		226	42.4
Never		118	22.1
Severity of depression symptoms	34		
No depression		177	34.0
Mild depression		173	33.2
Moderate depression		101	19.4
Severe depression		70	13.4

Self-evaluated health, feeling of loneliness, and the severity of depression symptoms as the SWB indicators (dependent variables)

### Objective sociodemographic or health-related characteristics as independent variables

The following objective factors were examined as potential predictors of SWB (Table 2). Sociodemographic characteristics included: age, gender, level of education measured with the number of years spent in education, place of residence (rural or urban), mode of living (alone vs. with family or as a resident in an institution), a high-stress situation in the recent past. Anthropomorphic measures such as body mass index (BMI) in kg/m^2^) and waist-to-hip ratio (WHR), were also taken into consideration.

**Table 2:** Characteristics of geriatric inpatients (*n* = 555)

Characteristics	Missing cases	Dichotomous variables		Continuous or polytomous variables				
	*n*	*n*	%	Min	Max	Median	Mean	±SD
Age	0			61	99	82.0	81.0	7.2
Male gender (yes)	0	152	27.4					
Number of years in education	4			0	25	7.0	8.7	4.3
Urban place of living (yes)	0	393	70.8					
Living alone (yes)	0	174	31.4					
High-stress situation in the recent past (yes)	9	189	34.6					
BMI (kg/m^2^)	47			14.5	56.8	27.8	28.7	6.0
Waist-to-hip ratio	38			0.56	1.2	0.91	0.91	0.08
Barthel index (0–100)	0			0	100	90	79,1	27.1
I-ADL (0–12)	0			0	12.0	8.0	6.8	4.1
AMTS (0–10)	9			0	10	8.0	7.1	2.7
Timed Up and Go (s)	110			5.2	97.1	15.5	19.4	12.5
Charlson comorbidity index (0–31)	0			2.0	21.0	7.0	7.1	2.6
Delirium (yes)	0	107	19.3					
Parkinson’s disease (yes)	0	91	16.1					
Hypertension (yes)	0	345	62.2					
Urine incontinence (yes)	0	267	48.1					
Fall(s) in recent year (yes)	6	225	41.0					
CRP (g/L)	2			0.56	1.2	0.91	0.91	0.08
Hemoglobin (g/dL)	4			6.2	17.6	12.9	12.7	1.6
Albumin (g/dL)	12			1.7	4.9	3.9	3.9	0.4

Potential objective predictors of the self-evaluated health, feeling of loneliness, and the severity of depression symptoms

*BMI* body mass index, *CRP* C-reactive protein blood test, *I-ADL* instrumental activities of daily living, *AMTS* abbreviated mental test scoring

Physical functional status was assessed using the Barthel index [25]—an ordinal rating scale for personal activities of daily living (P-ADL): feeding, bathing, grooming, dressing, bowel control, bladder control, toilet use, transfers (bed to chair), mobility, and stair use—where the total score ranges from a minimum of 0 (complete dependence) to a maximum of 100 (complete independence). Instrumental activity of daily living (I-ADL) was evaluated using the Duke OARS Assessment [26], where the summary score ranges from 0 (lowest function) to 12 (highest function). Six domains of functions were covered including preparing their own meals, going shopping, handling their own money, housework (cleaning floors and other tasks), using the telephone, and taking their own medicines. The patients’ cognitive status was assessed using the 10-item abbreviated mental test score [27] (the higher the score the better). Mobility was evaluated with the Timed Up and Go (TUG) test [28], which measures the seconds following the instruction to rise from the armchair and walk at a comfortable and safe walking speed to a line that is three meters away, turn around at the line, walk back to the chair, and sit down (the use of an assistance device was allowed if needed).

The degree of comorbidity was assessed with the Charlson comorbidity index [29] (CCI), where the score ranged from a minimum 0 to maximum of 31, depending on the presence of certain diseases (i.e., heart failure, dementia, diabetes, chronic obstructive pulmonary disease, chronic kidney disease, and cancer, among others) with assigned weights. Additionally, the presence of delirium, Parkinson’s disease, hypertension, fall(s) during the past year, and urinary incontinence were recorded. Biochemical findings such as C-reactive protein (in g/L), hemoglobin (in g/dL), and albumin (in g/dL) were also measured.

### Statistical analysis

First, univariate analyses were performed to identify the independent variables that exert a statistically significant influence on (1) the SEH, (2) the FoL, or (3) the SoDS. As all of these dependent measures are defined as ordinal categorical variables, the ordinal logistic regression (OLR) models were applied. The dependent variables were coded in the following way. For SEH, response of 1 means significantly worse than other people at the same age, 2 means moderately worse, 3 means moderately better, and 4 means significantly better. For FoL response of 1 means never feeling lonely, 2 means sometimes feeling lonely, and 3 means often feeling lonely. With regard to the severity of depression symptoms, an outcome of 1 means no depression, 2 means mild depression, 3 means moderate depression, and 4 means strong depression. A separate univariate OLR model was estimated for (1) the SEH, (2) the FoL, or (3) the SoDS and each of independent variables. Standard errors of parameter estimates were calculated using the robust Huber’s formula [30], and the significance level was set as a *p* value of <0.05.

Second, multiple ordinal logistic regression (OLR) models were estimated for each of dependent variables (SEH, FoL, and SoDS). The independent variables that did not prove to be significant in the univariate analyses were not included in the multivariate framework. The optimal OLR models, which contained the best subsets of independent variables and provided the highest explanatory power for the given dependent variables, were determined according to the Bayesian information criterion (BIC). BIC allows for balancing the goodness of fit of the statistical model against its complexity; moreover, in large samples BIC ideally corresponds to the candidate model that is rendered most plausible by the data [31, 32]. The proportional odds assumption was positively verified with the Brant test [33]. All statistical analyses were performed with the STATA software version 14.0 (StataCorp LP, College Station Texas).

## Results

### Univariate ordinal logistic regression analysis

The estimation results of the univariate OLR models are presented in Table 3.

**Table 3:** Impact of dependent variables on the self-evaluated health, feeling of loneliness, and the severity of depression symptoms–results from the univariate ordinal logistic regression (OLR) models

	Self-evaluated health		Feeling of loneliness		Severity of depression symptoms	
	Odds ratio	95 % CI	Odds ratio	95 % CI	Odds ratio	95 % CI
Age	1.02 (*p* = 0.062)	1.00, 1.05	**1.06** (*p* = 0.001)	1.01, 1.06	1.01 (*p* = 0.200)	0.99, 1.03
Male	1.37 (*p* = 0.077)	0.97, 1.95	**0.56** (*p* = 0.002)	0.39, 0.81	**0.57** (*p* = 0.001)	0.40, 0.80
Number of years spent in education	**1.08** (*p* < 0.000)	1.03, 1.13	**0.96** (0.026)	0.93, 0.99	**0.92** (*p* < 0.000)	0.89, 0.96
Urban place of residence	0.79 (*p* = 0.171)	0.56, 1.11	1.10 (*p* = 0.584)	0.77, 1.57	**1.77** (*p* = 0.001)	1.27, 2.46
Living alone	1.12 (*p* = 0.486)	0.80, 1.61	**2.99** (*p* < 0.000)	2.13, 4.19	1.38 (0.060)	0.99, 1.92
High-stress situation in the recent past	**0.65** (*p* = 0.017)	0.46, 0.92	**2.51** (*p* < 0.000)	1.78, 3.52	**1.95** (*p* < 0.000)	1.39, 2.72
BMI (kg/m^2^)	**0.95** (*p* < 0.000)	0.92, 0.97	1.02 (*p* = 0.094)	1.00, 1.05	1.02 (*p* = 0.202)	0.99, 1.05
Waist-to-hip ratio	0.24 (*p* = 0.226)	0.02, 2.44	0.15 (*p* = 0.097)	0.02, 1.41	1.20 (*p* = 0.185)	0.02, 2.17
Barthel index (0–100)	**1.02** (*p* < 0.000)	1.01, 1.02	**0.99** (*p* = 0.009)	0.99, 0.99	**0.98** (*p* < 0.000)	0.97, 0.99
I-ADL (0–12)	**1.13** (*p* < 0.000)	1.08, 1.17	**0.95** (*p* = 0.005)	0.91, 0.98	**0.90** (*p* < 0.000)	0.86, 0.94
AMTS (0–10)	1.02 (*p* = 0.507)	0.96, 1.09	0.96 (*p* = 0.155)	0.90, 1.02	**0.91** (*p* = 0.010)	0.85, 98
Timed Up and Go (s)	**0.97** (*p* = 0.001)	0.96, 0.99	**1.02** (*p* = 0.012)	1,00, 1.04	**1.03** (*p* = 0.001)	1.01, 1.04
Charlson comorbidity index (0–31)	**0.927** (*p* = 0.012)	0.87, 0.98	**1.12** (*p* < 0.000)	1.06, 1.19	**1.07** (*p* = 0.028)	1.01, 1.13
Delirium	1.09 (*p* = 0.67)	0.73, 1.63	1.45 (*p* = 0.067)	0.97, 2.16	1.32 (*p* = 0.229)	0.84, 2.10
Parkinson’s disease	**0.48** (*p* = 0.001)	0.31, 0.76	1.07 (*p* = 0.75)	0.70, 1.65	1.34 (*p* = 0.192)	0.86, 2.08
Hypertension	0.92 (*p* = 0.652)	0.63, 1.33	1.07 (*p* = 0.69)	0.75, 1.56	1.21 (*p* = 0.295)	0.84, 1.76
Urine incontinence (yes)	**0.66** (*p* = 0.012)	0.47, 0.91	**1.59** (*p* = 0.005)	1.15, 2.19	**1.92** (*p* < 0.000)	1.39, 2.65
Fall(s) in recent year (yes)	0.75 (*p* = 0.087)	0.54, 1.04	1.21 (*p* = 0.245)	0.88, 1.66	1.31 (*p* = 0.089)	0.96, 1.80
CRP (g/L)	**0.99** (*p* < 0.000)	0.98, 0.99	1.00 (*p* = 0.657)	0.99, 1.00	1.00 (*p* = 0.626)	0.99, 1,00
Hemoglobin (g/dL)	**1.14** (*p* = 0.013)	1.03, 1.27	0.91 (*p* = 0.059)	0.82, 1.00	0.98 (*p* = 0.668)	0.88, 1.09
Albumin (g/dL)	1.43 (*p* = 0.071)	0.97, 2.12	0.81 (*p* = 0.276)	0.56, 1.18	0.82 (*p* = 0.326)	0.55, 1.22

Values in bold indicate significant relationships (p < 0.05)

*BMI* body mass index, *CRP* C-reactive protein blood test, *I-ADL* instrumental activities of daily living, *AMTS* abbreviated mental test scoring

The increase in the number of years in education, hemoglobin (Hb), albumin level, Barthel index, and I-ADL scores significantly improved the SEH. On the other hand, a recent high-stress situation, urine incontinence, Parkinson’s disease, increase in the BMI, CRP, Charlson comorbidity index (CCI), and Timed Up and Go (TUG) test scores significantly diminished the SEH. With respect to FoL, living alone, urine incontinence, an increase in patient’s age, TUG, and CCI scores significantly enhanced the incidence of often feeling lonely. On the other hand, the increase in the number of years in education, Barthel index, and I-ADL scores proved to have a beneficial impact on social interactions and thus to alleviate the sense of loneliness. In terms of depression symptoms, an urban place of residence, a recent high-stress situation, urine incontinence, an increase in the CCI, and TUG scores significantly increased their severity. Being a male, as well as an increase in the number of years in education, Barthel index, I-ADL, and AMTS scores, on the other hand, proved to appreciably alleviate the depression symptoms in geriatric inpatients.

### Multiple ordinal logistic regression analysis

According to the BIC, the best combinations of independent variables that exert an independent impact on our response variables in the multiple ordinal logistic regression models were as follows:

1. The optimal model for SEH includes a high-stress situation in the recent past (OR^1^ 0.65; 95 % CI 0.44–0.94), level of education (OR 1.05; 95 % CI 1.0–1.1), I-ADL score (OR 1.11; 95 % CI 1.05–1.17), Hb level (OR 1.1; 95 % CI 0.98–1.25), BMI (OR 0.94; 95 % CI 0.92–0.97), and the speed of performing the Up & Go test (OR 2.08; 95 % CI 0.88–4.96). According to BIC, the reciprocal of the TUG score, hence the TUG speed (in m/s), turned out to provide much better explanatory power than the untransformed TUG variable.
2. The optimal model for FoL includes a high-stress situation in the recent past (OR 2.44; 95 % CI 1.71–3.50), living alone (OR 2.96; 95 % CI 2.08–4.20), level of education (OR 0.96; 95 % CI 0.92–0.99), the CCI (OR 1.12; 95 % CI 1.04–1.20), and the speed of performing the Up & Go test (OR 0.41; 95 % CI 0.20–0.83).
3. The optimal model for SoDS includes a high-stress situation in the recent past (OR 1.96; 95 % CI 1.39–2.75), level of education (OR 0.92; 95 % CI 0.89–0.96), the Barthel index (OR 0.98; 95 % CI 0.97–0.99), and the speed of performing the Up & Go test (OR 0.41; 95 % CI 0.16–0.99).

The obtained impact of the dichotomous independent variables can be interpreted as follows. The odds ratio for reporting significantly better versus moderately better, moderately worse, and significantly worse self-evaluated health status is 35 % lower for inpatients who experienced a high-stress situation in the recent past than for patients who did not. The OR for reporting often feeling lonely versus sometimes or never feeling lonely is nearly 2.5 times higher for inpatients reporting high-stress situation in the recent past. A high-stress situation in the recent past also induces symptoms of depression in geriatric inpatients. Of ultimate importance for the perceived sense of loneliness is the binary indicator of the living mode, because the odds ratio for reporting often feeling lonely versus sometimes or never feeling lonely is about three times higher for patients who live alone than for these who live with family or in institutions.

**Fig. 1:**
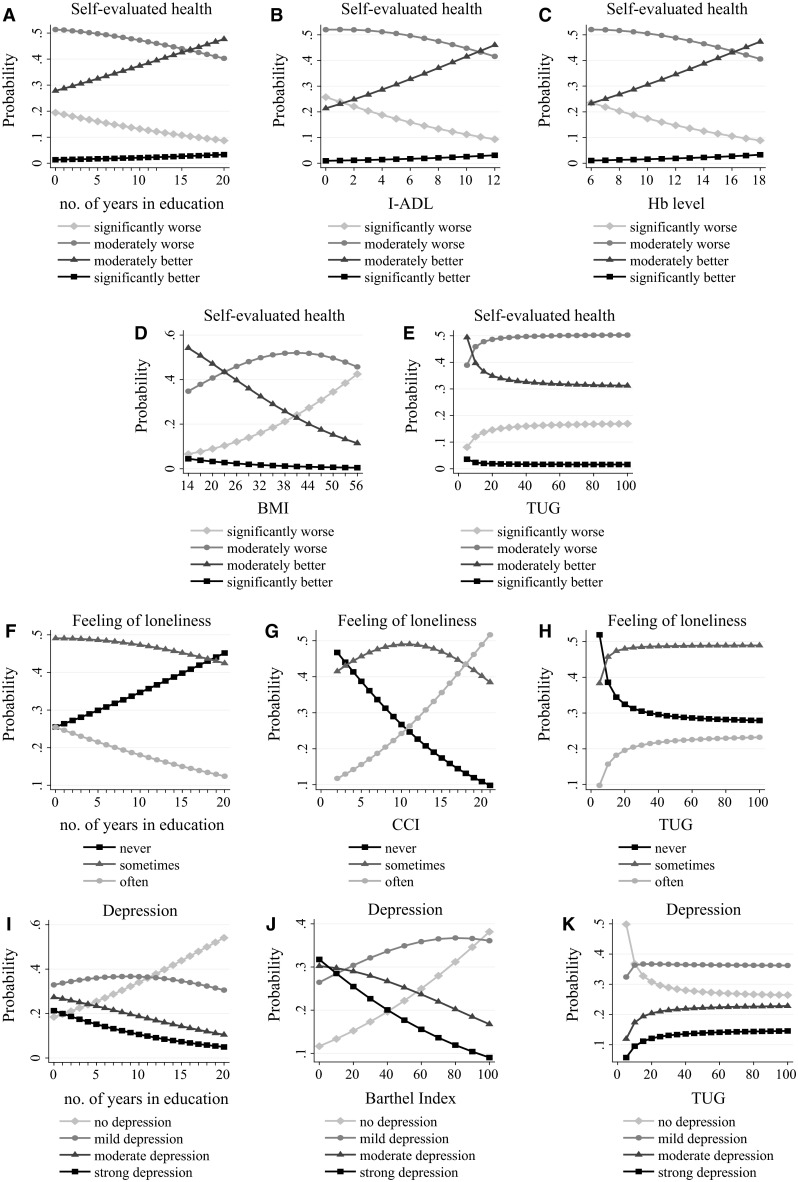
Predicted probabilities of individual responses in the self-evaluated health, feeling of loneliness, and the severity of depression symptoms. Marginal effects from the multiple ordinal logistic regression models. *I-ADL* instrumental activities of daily living, *Hb* Hemoglobin, *BMI* body mass index, *TUG* Timed Up and Go, *CCI* Charlson comorbidity index

Figure 1 depicts relationships between the best continuous (or ordinal) independent variables and the predicted probabilities of response variables’ categories obtained from the optimal multiple OLR models. For the SEH variable, level of education, the I-ADL score, and the Hb level have beneficial effects. For example, if the number of years in education is equal to 0, the probability that the average geriatric patient reports a moderately worse or significantly worse health status is equal to about 0.5 and 0.2, respectively (panel A). Both of these probabilities continue to decrease roughly linearly with the length of education, and for a patient with 20 years of education the probability drops to 0.4 and 0.1, respectively. The opposite relationship holds true for the probability that the average geriatric patient reports a moderately better or significantly better SEH. Panels B and C indicate a very similar influence with the I-ADL scores and the Hb levels on the SEH. In contrast to this, however, the BMI level and the outcome of the TUG test both exert a negative impact on the SEH status (panels D, E). The probabilities that average geriatric inpatients perceive their own health as moderately better or significantly better decline proportionally with the BMI level; however, the slope of the fall is much larger for the first than for the last. The probability of a patient reporting a significantly worse health rises more than proportionally with their BMI (panel D). The time needed to perform the Up & Go test has a nonlinear impact on the SEH status (panel E), and the probability that inpatients evaluate their own health as moderately better or significantly better decreases exponentially with the time needed for the Up &Go test to be completed. On the other hand, as the duration of performing the Up & Go exercise lengthens, there is a parallel inverted exponential growth of the probabilities that average inpatients perceive their own health status as moderately worse or significantly worse. The patterns obtained on the graph indicate that the marginal change in the outcome of the TUG exercise impacts the SEH status if the time needed to complete the test does not exceed 20 s. If it takes longer, however, its marginal impact on the SEH status is negligible.

Concerning the FoL (panels F–H), the level of education has a beneficial impact on the probability that average geriatric inpatients never feel lonely (panel F). For the number of education years equal to 0, the predicted probabilities of never feeling lonely or often feeling lonely are both nearly equal to 0.25. However, the probability lines diverge as the number of years spent in education increases, and for the patient with 20 years of schooling the probability of never feeling lonely reaches 0.45, whereas the probability of often feeling lonely drops to 0.12. An increase in the CCI, hence the level of comorbidity, severely increases the perceived sense of loneliness (panel G). The nonlinear, exponential relationship between the TUG variable and the FoL indicates that an increase in the TUG time from 5 to 30 s is met with a significant decrease in the probability of never feeling lonely (from 0.5 to 0.3), and a parallel moderate increase in the probability of sometimes feeling lonely or often feeling lonely (panel H). However, if the TUG changes by the same amount but begins with a higher value, for example from 40 to 65 s, its impact on a patient’s sense of loneliness is negligible.

The depression symptoms (panels I–K) are alleviated for the inpatients with longer education (panel I). The downward slope of the probability curves corresponding to strong depression or moderate depression are nearly the same. Similarly, improved mobility function relieves symptoms of depression (panels J and K). An increase in the Barthel index has a more-than-proportional effect on the probability that the assessment of depression is qualified as normal (panel J). A marginal increase in the duration of the TUG test enhances depression only if the test lasts less than about 20 s. In the case of a slower TUG performance, the change in the time needed for the exercise does not discriminate between the severity of a patient’s depression symptoms (panel K).

## Discussion

The main contribution of this analysis lies in definition of the best combination of objective, measurable explanatory factors that impact the SEH, FoL, and SoDS, as the three different dimensions in the complex concept of SWB in older persons [34, 35].

Although the initial set of sociodemographic or health-related characteristics of geriatric inpatients—the potential objective drivers of SWB—in our analysis was very broad, the best multivariate models describing SEH, FoL, and SoDS included only a handful of easily quantifiable explanatory variables. In terms of the SEH, the selection of BMI, I-ADL, and Hb levels correlated with the plethora of findings in the literature on older adults that was currently available. Our study confirms previous findings that high BMI, as a standard marker of being overweight, leads to lower self-evaluated health status in later life [36]. Similarly, our analysis confirms that lower I-ADL, the impaired ability to perform daily instrumental self-care activities, is also associated with poor self-evaluation of health [37]. Our results are also in line with previous findings that low Hb levels—a basic indicator of anemia—adversely affect the functional physical performance and outcomes of pre-frailty in older adults [38] as well as the predicted survival of geriatric inpatients [39]. The selection of the CCI as an optimal indicator of FoL correlates with the indication that a number of diseases can independently predict loneliness in the US population of older adults [40]. Interestingly, our study reveals that a unit increase in CCI is most closely associated with a change in the probability of registering two boundary categories of FoL. This means the probability of patients reporting that they never feel lonely declines, and there is a simultaneous rise in the probability that they always feel lonely. Our study also indicates that a higher Barthel index score exerts an independent positive impact on the SoDS, whereas the impaired ability to perform basic activities of daily living corresponds with a higher incidence of depression [41, 42].

The novelty of this study lies in the finding that controlling for the presence of high-stress situations which are well-known objective predictors of emotional state [43], two objective drivers—mobility performance assessed with the TUG test, and level of education—independently affect all three indicators of subjective well-being (SEH, FoL, and SoDS) in geriatric patients. An increase in education level is already known to improve self-rated health in later life [44], and the number of years spent in education is shown to enhance both life satisfaction and quality of life among older adults [45, 46]. However, our study takes a step forward by showing that the level of education independently contributes to a better SWB in the most comorbid elderly persons, alleviating their depression symptoms and perceived loneliness while improving their self-evaluated health status.

Impaired mobility is correlated with a worse quality of life in old age as measured with the LEIPAD questionnaire [47], and motor slowness has long been recognized as a symptom of clinical depression [48, 49]. Nevertheless, we documented the explicit nonlinear impact of the TUG score on the three different dimensions of SWB in geriatric inpatients after controlling for other sociodemographic or health-related factors. Although Anaby et al. [50] found that enhanced well-being might be derived from satisfaction with participation in daily activities rather than from success at accomplishing exercises, the results of this study strongly emphasize the underrated role of motor slowness (bradykinesia) as a universal marker for detection of a decreased state of mind among geriatric inpatients. Bradykinesia is an attribute of aging [51] that is caused by the gradual exhaustion of a patient’s homeostatic mechanisms [52] as well as a vast array of different physical dysfunctions resulting from comorbidities [53]. The results of our study show that the loss of mobility might account for most of the negative outcomes of old age, thus providing the best explanatory power for the subjective well-being of older inpatients with multiple chronic conditions.

Moreover, we show that the relationship between the time needed to perform the Up & Go test and the predicted probabilities of individual responses in self-evaluated health, feeling of loneliness, and severity of depression symptoms is highly nonlinear. This finding might have practical implications for everyday clinical practice. We show that, in practical terms, the marginal improvement in mobility (assessed with a small decrease in the time needed to perform the TUG test) has a beneficial impact on perceived well-being only in inpatients who complete the Up & Go task in less than 20 s. If the overall duration of the TUG exercise is longer than 20 s, the small change in time needed to complete the test has a negligible patient’s subjective well-being. However, it might help to stratify inpatients into different but stable levels of subjective well-being, i.e., response modalities for the self-evaluated health, feeling of loneliness, and the severity of depression symptoms.

This analysis has some limitations that must be addressed. First, it is important to note that the cross-sectional design of the study could have an effect on the evaluation of causal relationships between certain variables. For example, physical slowness, represented by a high TUG score, may be treated as both a cause and an effect of depression. Analogously, comorbidity may increase self-perceived loneliness, while the converse may also be true because there is evidence that social isolation leads to increased comorbidity [54].

Second, the SEH and FoL variables were both measured with only one question; therefore, inpatients’ answers could have been affected by a transitory or accidental state of mind. Additionally, the question measuring SEH had only four possible answers in comparison with five possible answers recommended by the World Health Organization, i.e., very good, good, moderate, bad, or very bad [55]. Nevertheless, population-based studies have indicated that different versions of self-rated health questions result in parallel assessments of the same latent health variable [56, 57].

Lastly, our sample of geriatric inpatients does not allow us to apply these results to the general population of older adults. However, this study reveals novel findings for older adults who are comorbid and frail.

This study provides new insights into how easily measurable, objective characteristics of geriatric patients contribute to the selected aspects of SWB (i.e., SEH, FoL, and SoDS). The level of education and the performance-based measure of mobility (Timed Up and Go test) also offer substantial information on the predicted emotional states of geriatric inpatients after controlling for age, gender, number of different illnesses and disabilities, and other sociodemographic and health-related characteristics. The results of our study may serve as a pragmatic guide for geriatric medical practitioners, encouraging them to promote suitably tailored physical activity among older adults.

## Footnotes

1 OR is the odds ratio for the ordered logistic regression model.
